# Do health preferences differ among Asian populations? A comparison of EQ-5D-5L discrete choice experiments data from 11 Asian studies

**DOI:** 10.1007/s11136-021-03075-x

**Published:** 2022-02-18

**Authors:** Zhihao Yang, Fredrick Dermawan Purba, Asrul Akmal Shafie, Ataru Igarashi, Eliza Lai-Yi Wong, Hilton Lam, Hoang Van Minh, Hsiang-Wen Lin, Jeonghoon Ahn, Juntana Pattanaphesaj, Min-Woo Jo, Vu Quynh Mai, Jan Busschbach, Nan Luo, Jie Jiang

**Affiliations:** 1 Health Services Management Department, Guizhou Medical University, Guiyang, China; 2 Faculty of Psychology, Universitas Padjadjaran, Jatinangor, Indonesia; 3 School of Pharmaceutical Sciences, Universiti Sains Malaysia, Gelugor, Malaysia; 4 Department of Drug Policy and Management, Faculty of Psychology, Graduate School of Pharmaceutical Sciences, University of Tokyo, Tokyo, Japan; 5 Centre for Health Systems and Policy Research, JC School of Public Health and Primary Care, Faculty of Medicine, The Chinese University of Hong Kong, Hong Kong Special Administrative Region, China; 6 Institute of Health Policy and Development Studies, National Institutes of Health, University of the Philippines Manila, Manila, Philippines; 7 Center for Population Health Sciences, Haoni University of Public Health, Hanoi, Vietnam; 8 School of Pharmacy and Graduate Institute, China Medical University, Taichung, Taiwan; 9 Department of Health Convergence, Ewha Womans University, Seoul, South Korea; 10 Ministry of Public Health, Nonthaburi, Thailand; 11 Department of Preventive Medicine, University of Ulsan College of Medicine, Seoul, South Korea; 12 Department of Psychiatry, Section Medical Psychology and Psychotherapy, Erasmus MC University Medical Center, Rotterdam, The Netherlands; 13 Saw Swee Hock School of Public Health, National University of Singapore, Singapore, Singapore; 14 School of Pharmacy, Jian University, No. 601 Huangpudadaoxi, Guangzhou, China

**Keywords:** EQ-5D, DCE, Asian, Health preference

## Abstract

**Introduction:**

Many countries have established their own EQ-5D value sets proceeding on the basis that health preferences differ among countries/populations. So far, published studies focused on comparing value set using TTO data. This study aims to compare the health preferences among 11 Asian populations using the DCE data collected in their EQ-5D-5L valuation studies.

**Methods:**

In the EQ-VT protocol, 196 pairs of EQ-5D-5L health states were valued by a general population sample using DCE method for all studies. DCE data were obtained from the study PI. To understand how the health preferences are different/similar with each other, the following analyses were done: (1) the statistical difference between the coefficients; (2) the relative importance of the five EQ-5D dimensions; (3) the relative importance of the response levels.

**Results:**

The number of statistically differed coefficients between two studies ranged from 2 to 16 (mean: 9.3), out of 20 main effects coefficients. For the relative importance, there is not a universal preference pattern that fits all studies, but with some common characteristics, e.g. mobility is considered the most important; the relative importance of levels are approximately 20% for level 2, 30% for level 3, 70% for level 4 for all studies.

**Discussion:**

Following a standardized study protocol, there are still considerable differences in the modeling and relative importance results in the EQ-5D-5L DCE data among 11 Asian studies. These findings advocate the use of local value set for calculating health state utility.

**Supplementary Information:**

The online version contains supplementary material available at 10.1007/s11136-021-03075-x.

## Summary

The EQ-5D DCE data provided an ideal data source to understand the health preference variations in Asia. By comparing the modeling results, the relative importance of dimensions and levels, we found Asian regions have diverse health preferences.

## Introduction

EQ-5D is a generic preference-based health-related quality of life (HRQoL) questionnaire that is widely used around the world [1, 2]. When value sets are available, EQ-5D data can be converted to health utility [2]. Many countries have established their own EQ-5D value sets proceeding on the basis that health preferences differ among countries/populations [3, 4]. Indeed, studies have found differences between value sets [3, 5, 6]. In developing the value sets of three-level version of EQ-5D (EQ-5D-3L), published studies differed in terms of design, data collection protocol and the choice of model. By comparing these value sets, Norman et al. concluded that these variations in methods could obscure true differences in values [6]. For the latest five-level version of EQ-5D (EQ-5D-5L), the EuroQol Group developed a standardized protocol for data collection in valuation studies, which is named the EuroQol valuation technology protocol (EQ-VT) [7–9].

With application of the EQ-VT, EQ-5D-5L valuation data can be exploited to study whether important differences in health preferences across populations exist, as the method variations observed in the 3L studies are minimized. The EQ-VT data collection protocol uses both time trade-off (TTO) and discrete choice experiment (DCE) as preference elicitation methods [7]. Currently, all comparison studies of EQ-5D value sets only used the TTO data from the valuation studies [5, 10, 11]. This is partially because the TTO data is considered as the primary preference source in the EQ-VT protocol and some studies estimated their value set using TTO data only, for example, China and South Korea [12, 13]. So far, the DCE data collected using the EQ-VT protocol has not been utilized for the purpose of identifying preference differences across studies. While the TTO valuation data could be subject to interviewer effects as the task relies on the good performance of the interviewers [14], there is minimal interviewer effect for the DCE data.

As a preference elicitation method, DCE has been increasingly used in health preference studies [15]. Based on random utility theory, DCE is designed to ask respondents to choose a preferred multi-dimensional health state from two or more alternatives. The ordinal preference data can be modeled to predict health utility on a latent scale [16]. This means that the coefficients of DCE are not directly comparable across studies and most studies assessed their difference by calculating and comparing the relative importance of five health dimensions [17, 18].

Further, differences in health preferences among Asian populations are not well understood. By comparing the multiplicative model coefficients of the EQ-5D-5L TTO valuation data from seven Asian studies, Wang et al. noticed that there was no consensus about the rank ordering of the five dimensions [10]. Additionally, statistical test suggested most coefficients differed among Asian studies. In the study of Roudijk et al., the authors found that cultural variables (i.e. traditional/rational-secular, survival/self-expression) did not explain the variations of value differences (defined as utility differences between the mild and severe states) among EQ-5D valuation studies, including 10 Asian studies [11]. As stated before, these studies only explored the TTO valuation data.

Following the EQ-VT protocol, 11 studies (China, Indonesia, Japan, South Korea, Malaysia, Singapore, Thailand, Philippines, Vietnam, Hong Kong, Taiwan) have been completed in Asia. Of those studies, China and South Korea did not use the DCE data to model the value sets, and Singapore and Philippines have not yet published their value sets. The rest of the studies modeled the DCE data and TTO data jointly. Notably, no study has compared DCE-derived preference data among Asian populations. Given all studies used the standardized EQ-5D-5L instrument, DCE experimental design, and data collection protocol, it is possible to explore the variations of health preferences in Asia. We hypothesized that the health preferences differed in Asian populations. If this is true, the results of this study could further support the establishment of national/regional value sets for better guidance of health care decision-making and resource allocation rather than using a unified value set designed merely for the continent. In this study, we aim to understand the similarities and differences in Asians’ preferences for EQ-5D-5L health states in 11 Asian DCE datasets collected as part of EQ-5D-5L valuation studies.

## Methods

### EQ-5D-5L questionnaire and 11 Asian valuation studies

The EQ-5D-5L measures HRQoL using five dimensions (mobility, self-care, usual activities, pain/discomfort, anxiety/depression), and under each dimension, there are five levels of severity (no problems, slight problems, moderate problems, severe problems, extreme problems/unable to). In total, the EQ-5D-5L defines 5^5^ = 3,125 health states [19]. We obtained the DCE data from the principal investigators of 11 Asian EQ-5D-5L valuation studies, namely China [12], Indonesia [20], Japan [21], South Korea [13], Malaysia [22], Singapore, Thailand [23], the Philippines, Vietnam [24], Hong Kong [25], and Taiwan [26]. The language(s) being used in each study is the official language and/or dialects that are spoken mostly by the population (see Table 1).

**Table 1:** Basic information of the 11 studies

Study	Sample size*	Sample age range	Sampling strategy	Sampling determinants	Language(s)**	Data collection time
China	1300	18–85	Quota	Metropolitan areas, age, sex, and educational attainment	Mandarin	2012
Indonesia	1056	17–75	Quota	Urbanicity, sex, age, educational attainment, religion, and ethnicity	Bahasa Indonesia	2015–2016
Japan	1262	20–69	Stratified	Region, sex, and age	Japanese	2014
South Korea	1080	19–87	Quota	Region, sex, age, and educational attainment	Korean	2013
Malaysia	1143	18–88	Quota	Region, urbanicity, sex, age, and ethnicity	Malay and English	2016
Singapore	1849	10–99	Quota	Age, gender and ethnicicty	English and Mandarin	2014–2015
Thailand	1209	18–84	Quota	Region, age and sex	Thai	2013–2014
Philippines	1000	18–80	Quota	Age, sex, region, urbanicity, educational attainment, income, and ethno-linguistic	Tagalog, Cebuano, and English	2017
Vietnam	1201	18–88	Quota	Region, urbanicity, age, and sex	Vietnamese	2017
Hong Kong	1014	18–88	Quota	Sex, age, and educational attainment	Cantonese	2014–2015
Taiwan	1000	20–90	Quota	Region, age, sex, and educational attainment	Traditional Chinese	2017

*The final sample size for estimating the value set may differ with the sample size shown above

**The language(s) being used in each study is the official language and/or dialects that are spoken mostly by the population

### DCE design and tasks

**Fig. 1:**
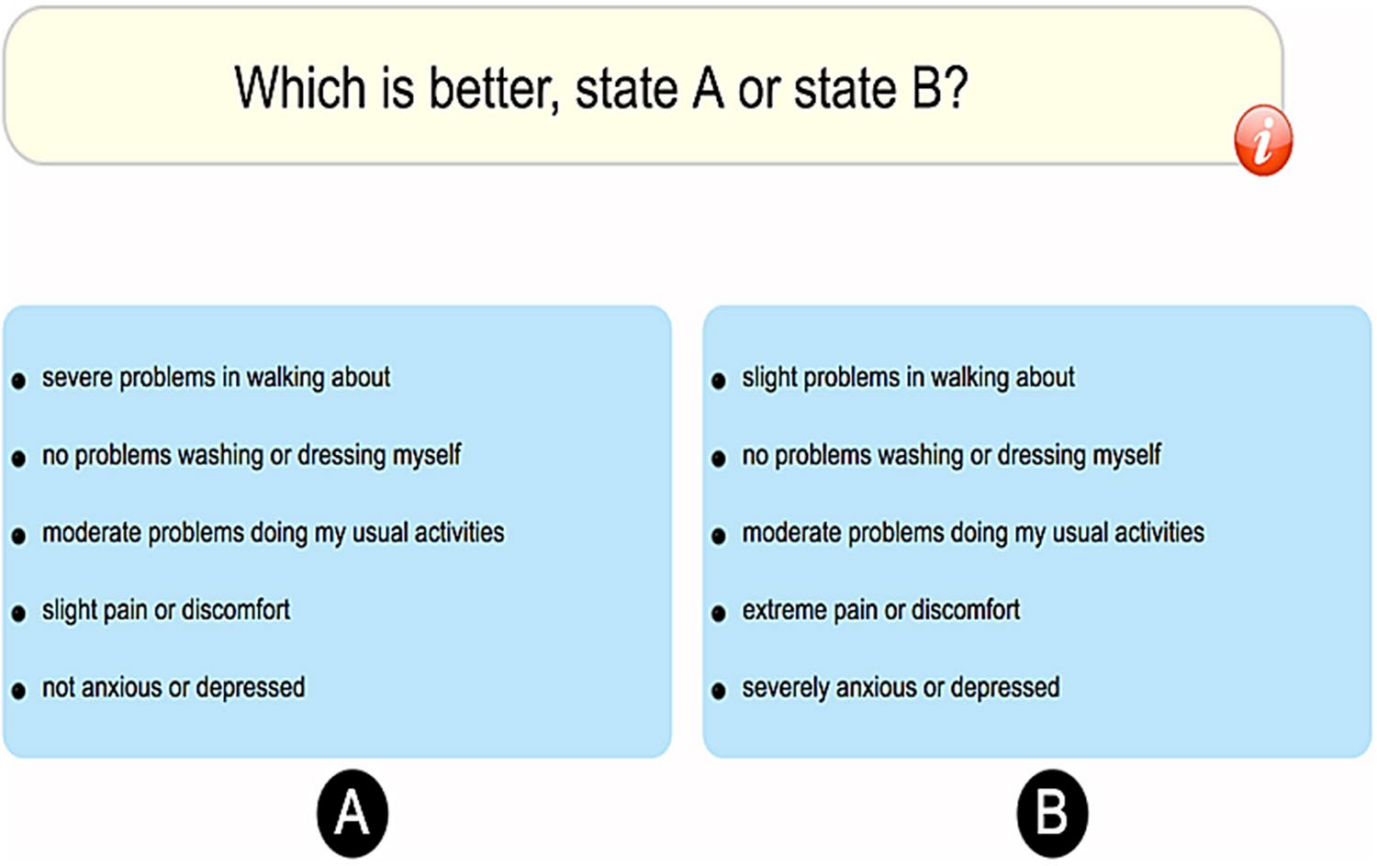
An example of Discrete Choice Experiments (DCE) in English

All Asian studies included in this study used the standard EQ-VT protocol for data collection [9, 27]. In general, the DCE design of the EQ-VT protocol consisted of a total of 196 pairs of health states including 186 pairs generated from a Bayesian efficient design algorithm and 10 pairs of mild states [27]. The priors for the Bayesian efficient design algorithm were extracted from a main effects model of an EQ-5D-3L DCE study [28]. The detailed experimental design development process and considerations were described in Oppe et al. [27]. The 196 pairs of EQ-5D-5L health states were distributed over 28 blocks, each consisting of 7 pairs of health states with similar severity. No dominant pairs were included. [27]. In each study, each respondent was assigned one block of DCE tasks to complete. The 7 pairs were presented in random order, and the right-left presentation of the two health states was also randomized [8]. Figure 1 shows the screenshot of one DCE task in EQ-VT software.

### Data collection

Following the EQ-VT protocol, all respondents were interviewed face-to-face by a trained interviewer using the EQ-VT software. The data collection included four sections: The first section was for respondents to report their own health using the EQ-5D-5L descriptive system and the EQ-VAS. In the second section, respondents valued 10 different EQ-5D-5L health states using the composite time trade-off (cTTO) [8]. In the third section, respondents completed 7 pairs of EQ-5D-5L discrete choice tasks [27]. Finally, respondents reported their socio-economic and other background characteristics. We used the DCE data obtained from the third section for the analysis.

### Analysis

To understand how the health preferences are different/similar with each other, the following analyses were done: (1) the statistical difference between the coefficients; (2) the relative importance of the five EQ-5D dimensions; (3) the utility decrements between each of the response levels.

For modeling, a 20-parameter main-effects mixed logit model was fitted for each study. In this model (Formula 1), utility was explained by 20 dummy variables and was on a latent scale (referred as latent utility). For each dimension (MO for mobility, SC for self-care, UA for usual activities, PD for pain/discomfort, AD for anxiety/depression), 4 dummy variables were used to represent the departure from level 1 to the other 4 levels, e.g. MO_3_ was 1 if the health state being valued had “moderate problems with mobility” and 0 for any other level of mobility [29]. In addition, a heteroscedastic conditional model was also fitted for each study [30]. The major difference between the heteroscedastic conditional logit model and the mixed logit model is that the heteroscedastic conditional logit model accounted for the heterogeneity in error variance and the mixed logit model accounted for the preference heterogeneity among respondents.

1 $$ Latent\;utility = \beta_{1} MO_{2} + \beta_{2} MO_{3} + \beta_{3} MO_{4} + \beta_{4} MO_{5} + \beta_{5} SC_{2} + \ldots + \beta_{20} AD_{5} + \varepsilon $$

Next, the statistic difference between two studies’ coefficients were explored using a pairwise comparison. For each pair, a dummy variable was generated as 0 for one study’s data and as 1 for the other. Next, a 20-parameter main-effects model plus 20 interaction terms was fitted for all two-by-two study combinations (see Formula 2). In this model with interaction terms, a significant interaction term suggests that the coefficient is statistically different between two studies. The number of statistically differed coefficients were summarized for each study pair. Notably, the coefficient of a significant interaction term may not exceed the minimal important difference (MID) on the utility scale [31].

2 $$ Latent\;utility = \beta_{1} MO_{2} + \beta_{2} MO_{3} + \beta_{3} MO_{4} + \beta_{4} MO_{5} + \beta_{5} SC_{2} + \ldots + \beta_{20} AD_{5} + \beta_{21} MO_{2} *study\;dummy + \beta_{22} MO_{3} *study \, dummy + \beta_{23} MO_{4} *study\;dummy \ldots + \beta_{40} AD_{5} *study \, dummy + \varepsilon $$

Using the mixed effect logit model results (Formula 1), the relative importance of dimensions and levels were estimated for each study [17, 18, 32]. The relative importance of the five dimensions were calculated in two steps. First, the dimension-level coefficient was divided by the mean of the same level from all the dimensions. For example, the adjusted coefficient for mobility level 3 was obtained by the MO_3_ coefficient divided by the sum of all level 3 coefficients for each dimension aMO_3_ = MO_3_/(MO_3_ + SC_3_ + UA_3_ + PD_3_ + AD_3_). This step resulted in adjusted coefficients for the last four levels (level 1 is the reference level) of every dimension. Second, the means of all adjusted coefficients for each dimension were calculated. Continuing the mobility example, the relative dimension importance of mobility for a study would be estimated as (aMO_2_ + aMO_3_ + aMO_4_ + aMO_5_)/4.

The relative importance of levels was also obtained in two steps: first, the sum of each level coefficient from all dimensions was calculated. Second, the sum of each level coefficient was divided by the sum of level 5 coefficients: e.g. the relative importance of level 2 was the sum coefficient of level 2 divided by the sum coefficient of level 5. In practice, relative importance for level 2 sum for a study would be calculated as follows: (MO_2_ + SC_2_ + UA_2_ + PD_2_ + AD_2_)/ (MO_5_ + SC_5_ + UA_5_ + PD_5_ + AD_5_). The relative importance results were summarized across 11 studies and two figures were plotted, one for the relative importance of the dimensions and one for the relative importance of levels (see Online Appendix 1 for the calculation of the relative important). If five dimensions are equally weighted by a population, all five dimensions should have a relative importance of 0.20 (i.e. 1 divided by 5). The relative importance of levels is interpreted as the percentage of the weight attached to level five problems. The 95% confidence intervals of relative importance were calculated using the Delta method (see Online Appendix 2 for an example STATA code). Analyses were performed using STATA 14 (Stata Corp LLC) [33].

## Results

### Data descriptions

Table 1 summarizes the key information from the 11 valuation studies. Based on the EQ-VT protocol, all studies recruited at least 1000 respondents. Quota sampling was the most used sampling strategy, but the quota differed. All studies were conducted between 2012 and 2017.

### Modeling results

Table 2 shows the mixed logit modeling results. All coefficients for all studies were significant at 0.05 level except for the second level of usual activities in Taiwan. Vietnam and Philippines each had 1 and 2 inconsistent coefficients, respectively. Three inconsistencies occurred on the third level of self-care, mobility, and usual activities, respectively. Within each study, the standard errors of the coefficients generally increased with severity levels. Table 3 shows the number of coefficients that differed statistically between two studies. Overall, 9.3 out of 20 coefficients differed among studies. Almost all studies had at least 5 coefficient differences with others except for Taiwan versus Hong Kong, Taiwan versus Malaysia. Malaysia and Singapore differed the most with 16 statistically different coefficients. An example of this comparison between China and Indonesia can be found in Online Appendix 3.

**Table 2:** The results of the mixed logit model for 11 studies

	China	Indonesia	Japan	South Korea	Malaysia	Singapore	Thailand	Philippines	Vietnam	Hong Kong	Taiwan
Mean*											
mo2	− 0.522, 0.059	− 0.625, 0.069	− 0.572, 0.059	− 0.711, 0.066	− 0.643, 0.069	− 0.507, 0.049	− 0.560, 0.067	− 0.702, 0.068	− 0.755, 0.069	− 0.696, 0.071	− 0.720, 0.073
mo3	− 0.903, 0.075	− 1.037, 0.088	− 0.713, 0.073	− 0.924, 0.085	− 1.004, 0.089	− 0.678, 0.061	− 0.591, 0.083	**− 0.629**, 0.081	− 0.845, 0.084	− 1.079, 0.089	− 1.086, 0.094
mo4	− 1.579, 0.086	− 1.960, 0.105	− 1.336, 0.080	− 1.584, 0.095	− 2.087, 0.113	− 1.479, 0.070	− 1.694, 0.102	− 1.582, 0.094	− 1.923, 0.103	− 2.062, 0.107	− 2.089, 0.113
mo5	− 2.366, 0.110	− 3.136, 0.155	− 1.980, 0.102	− 3.078, 0.150	− 3.089, 0.155	− 2.194, 0.089	− 3.300, 0.149	− 2.251, 0.122	− 3.367, 0.151	− 3.001, 0.143	− 2.996, 0.145
sc2	− 0.243, 0.065	− 0.463, 0.076	− 0.387, 0.066	− 0.194, 0.072	− 0.573, 0.079	− 0.308, 0.054	− 0.521, 0.078	− 0.559, 0.074	− 0.461, 0.079	− 0.376, 0.078	− 0.330, 0.082
sc3	− 0.524, 0.073	− 0.653, 0.084	− 0.501, 0.073	− 0.258, 0.080	− 0.720, 0.091	− 0.527, 0.060	− 0.534, 0.085	− 0.607, 0.080	**− 0.449**, 0.085	− 0.503, 0.084	− 0.587, 0.090
sc4	− 1.119, 0.080	− 1.057, 0.090	− 0.881, 0.075	− 0.606, 0.083	− 1.798, 0.106	− 1.162, 0.067	− 1.490, 0.099	− 1.438, 0.090	− 1.232, 0.092	− 1.411, 0.096	− 1.491, 0.101
sc5	− 1.531, 0.086	− 1.542, 0.097	− 1.251, 0.078	− 1.062, 0.086	− 2.165, 0.118	− 1.497, 0.071	− 2.005, 0.109	− 1.608, 0.090	− 1.964, 0.108	− 1.763, 0.104	− 1.949, 0.113
ua2	− 0.352, 0.062	− 0.382, 0.073	− 0.376, 0.061	− 0.266, 0.069	− 0.294, 0.073	− 0.195, 0.051	− 0.312, 0.073	− 0.474, 0.070	− 0.290, 0.073	− 0.248, 0.074	− ***0.106***, 0.075
ua3	− 0.449, 0.072	− 0.654, 0.083	− 0.506, 0.071	− 0.338, 0.080	− 0.376, 0.086	− 0.286, 0.058	− 0.379, 0.086	**− 0.473**, 0.078	− 0.412, 0.080	− 0.434, 0.083	− 0.292, 0.089
ua4	− 0.986, 0.076	− 1.391, 0.094	− 1.041, 0.077	− 0.917, 0.086	− 1.220, 0.096	− 0.732, 0.059	− 1.130, 0.088	− 1.128, 0.086	− 1.368, 0.092	− 1.064, 0.087	− 1.340, 0.098
ua5	− 1.648, 0.087	− 1.800, 0.100	− 1.452, 0.087	− 1.627, 0.102	− 1.577, 0.100	− 0.952, 0.063	− 2.016, 0.110	− 1.409, 0.091	− 2.420, 0.118	− 1.409, 0.095	− 1.877, 0.111
pd2	− 0.521, 0.066	− 0.399, 0.076	− 0.316, 0.066	− 0.374, 0.074	− 0.691, 0.080	− 0.302, 0.053	− 0.345, 0.077	− 0.590, 0.073	− 0.825, 0.078	− 0.491, 0.079	− 0.518, 0.079
pd3	− 0.758, 0.073	− 0.449, 0.079	− 0.504, 0.070	− 0.576, 0.080	− 0.789, 0.090	− 0.335, 0.057	− 0.390, 0.082	− 0.624, 0.079	− 1.300, 0.092	− 0.746, 0.086	− 0.698, 0.089
pd4	− 1.669, 0.084	− 0.875, 0.087	− 1.041, 0.074	− 1.451, 0.094	− 1.987,0.111	− 1.187, 0.066	− 1.509, 0.098	− 1.562, 0.095	− 2.108, 0.106	− 1.643, 0.101	− 1.835, 0.109
pd5	− 2.101, 0.101	− 1.151, 0.090	− 1.747, 0.094	− 2.291, 0.131	− 2.965, 0.153	− 1.798, 0.080	− 2.007, 0.115	− 1.777, 0.102	− 2.992, 0.136	− 1.930, 0.115	− 3.022, 0.159
ad2	− 0.256, 0.068	− 0.200, 0.080	− 0.292, 0.066	− 0.194, 0.075	− 0.506, 0.082	− 0.234, 0.057	− 0.343, 0.081	− 0.285, 0.075	− 0.363, 0.077	− 0.368, 0.082	− 0.211, 0.084
ad3	− 0.715, 0.071	− 0.534, 0.080	− 0.623, 0.070	− 0.428, 0.080	− 0.773, 0.086	− 0.454, 0.057	− 0.624, 0.081	− 0.467, 0.076	− 0.830, 0.083	− 0.637, 0.083	− 0.794, 0.089
ad4	− 1.485, 0.086	− 1.053, 0.086	− 1.156, 0.077	− 1.029, 0.089	− 1.826, 0.111	− 1.184, 0.065	− 1.855, 0.104	− 1.082, 0.087	− 1.376, 0.093	− 1.500, 0.098	− 1.756, 0.111
ad5	− 1.850, 0.092	− 1.441, 0.096	− 1.712, 0.092	− 1.260, 0.096	− 2.486, 0.125	− 1.515, 0.070	− 2.584, 0.123	− 1.065, 0.088	− 1.801, 0.103	− 1.768, 0.106	− 2.269, 0.117
SD#											
mo2	**0.008**	**− 0.153**	**0.014**	**0.031**	**− 0.126**	**− 0.126**	**− 0.038**	**0.141**	**0.068**	**0.041**	**− 0.018**
mo3	**0.341**	**0.080**	0.494	− 0.408	**0.081**	0.359	**0.144**	**− 0.232**	**0.113**	**− 0.040**	**0.027**
mo4	0.603	0.480	**0.318**	0.568	0.710	0.787	0.823	**0.344**	0.657	− 0.598	− 0.554
mo5	1.059	1.280	0.968	1.644	1.461	1.101	1.360	1.127	1.340	1.117	0.891
sc2	**− 0.016**	− 0.443	**− 0.204**	**− 0.003**	**0.204**	**− 0.193**	− 0.392	**0.286**	− 0.608	**0.246**	**0.219**
sc3	**0.110**	**− 0.186**	**− 0.214**	− 0.367	− 0.535	**0.056**	**− 0.285**	**0.182**	0.401	**− 0.244**	**0.206**
sc4	**− 0.239**	0.487	**0.174**	**− 0.232**	**− 0.150**	0.546	− 0.451	**− 0.299**	**− 0.279**	**0.438**	**0.257**
sc5	0.541	0.738	**0.222**	**0.246**	0.827	0.754	0.462	0.364	0.976	0.832	0.524
ua2	**0.109**	**− 0.290**	**− 0.071**	**− 0.093**	**0.223**	**− 0.031**	0.452	**0.007**	0.487	**0.079**	**0.201**
ua3	**− 0.293**	**− 0.208**	**0.201**	**− 0.232**	0.385	**0.106**	− 0.657	**0.065**	**0.234**	**0.122**	**− 0.043**
ua4	0.472	0.592	− 0.404	− 0.463	0.462	**− 0.054**	**0.238**	− 0.602	− 0.470	0.455	**0.198**
ua5	0.642	0.816	0.919	0.937	**0.113**	0.542	0.739	0.491	0.989	**0.156**	0.799
pd2	**− 0.210**	**− 0.229**	**0.214**	**0.037**	**0.300**	**− 0.075**	**− 0.336**	**0.075**	**− 0.044**	− 0.385	**0.095**
pd3	**− 0.007**	**0.070**	**0.176**	**− 0.094**	**− 0.294**	**− 0.127**	**0.245**	**0.051**	**0.262**	**0.034**	**0.069**
pd4	**0.263**	− 0.640	**0.112**	**0.425**	0.888	0.567	− 0.937	0.451	− 0.527	− 0.491	0.700
pd5	1.055	**− 0.340**	0.851	1.782	1.482	1.186	1.230	− 0.743	− 0.974	0.937	1.891
ad2	**0.203**	0.680	**− 0.144**	0.345	**− 0.137**	0.489	0.518	**− 0.355**	**0.086**	− 0.621	− 0.361
ad3	**0.130**	**0.254**	**− 0.159**	**0.080**	**0.163**	**− 0.225**	**− 0.101**	**0.151**	**− 0.137**	**− 0.031**	**0.231**
ad4	− 0.361	**0.325**	**0.028**	0.284	**0.292**	**0.210**	0.458	0.428	**0.047**	**0.388**	**− 0.352**
ad5	− 0.559	**0.169**	− 0.797	− 0.564	**− 0.134**	0.534	− 0.648	− 0.557	0.411	− 0.722	**0.236**
Number of observations	18,200	14,756	16,838	15,120	16,002	25,886	16,898	14,000	16,814	14,196	14,000
Log-likelihood	− 4666.48	− 3905.76	− 4602.26	− 4021.33	− 3711.54	− 7088.60	− 4027.34	− 3756.50	− 3942.43	− 3587.43	− 3204.25
AIC	9412.97	7891.52	9284.53	8122.67	7503.08	14,257.19	8134.69	7592.99	7964.86	7254.85	6488.50
BIC	9725.34	8195.49	9593.78	8427.62	7810.29	14,583.65	8444.08	7894.87	8274.05	7557.28	6790.37

*For the ‘Mean’ coefficients: Italic and bold font suggests the coefficient is not significant at 0.05 level; bold font suggests the coefficient is inconsistent

#For the ‘SD’ coefficients: Bold font suggests not significant at 0.05 level. The direction of the SD does not have meaning

**Table 3:** Number of coefficients differed statistically between two studies

	China	Indonesia	Japan	Korea	Malaysia	Singapore	Thailand	Philippines	Vietnam	Hong Kong
Indonesia	9									
Japan	8	8								
Korea	9	12	10							
Malaysia	7	12	9	13						
Singapore	13	10	8	12	16					
Thailand	9	10	7	8	8	8				
Philippine	8	10	8	10	10	12	8			
Vietnam	9	12	10	14	6	12	11	8		
Hong Kong	5	9	7	12	5	13	6	7	8	
Taiwan	8	10	11	12	2	15	6	10	10	3

Compared with the mixed logit model results, the heteroscedastic conditional logit model improved the non-significance for Taiwan but did not improve the coefficient inconsistency for Philippine and Vietnam. Furthermore, this model resulted one non-significant coefficient for South Korea and one inconsistency for Thailand. The heteroscedastic conditional modeling results can be found in Online Appendix 4.

### Relative weight results

**Fig. 2:**
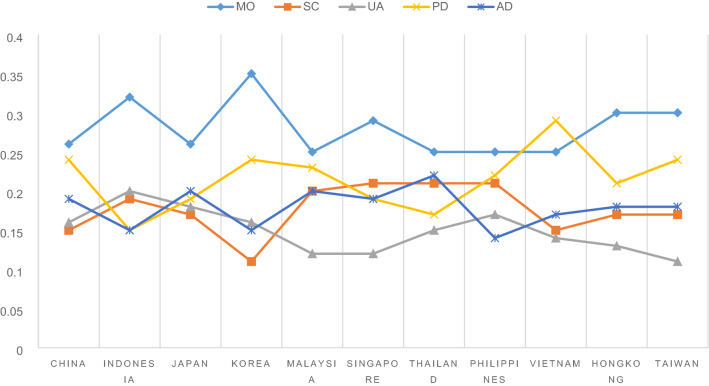
Relative importance of five dimensions

Table 4 shows the relative importance and their 95% confidence intervals of 11 studies. Figure 2 shows a universal rank order does not exist across 11 Asian populations. Mobility was the most important dimension for every study except for Vietnam. The lowest important dimension was either usual activities or self-care except for Philippines and Indonesia. Notably, these two functional dimensions had similar weights in China, Indonesia, Japan and Vietnam, and only Korea had larger relative weight for usual activities. Pain/discomfort was the second most important dimension for 6 studies, and it was valued higher than or equal to anxiety/depression in almost all studies except for Thailand. Singapore, Japan, Philippines, and Indonesia placed similar weights on pain/discomfort and anxiety/depression. The sum of the first three functional dimensions were larger than the sum of the two symptom dimensions across all studies.

**Table 4:** Relative importance of 11 studies, mean (95% confidence intervals)

	China	Indonesia	Japan	Korea	Malaysia	Singapore	Thailand	Philippines	Vietnam	Hongkong	Taiwan
MO	0.256 (0.231,0.282)	0.317 (0.287,0.347)	0.258 (0.230,0.286)	0.347 (0.310,0.384)	0.249 (0.227,0.272)	0.290 (0.261,0.318)	0.250 (0.220,0.280)	0.251 (0.224,0.278)	0.252 (0.230,0.274)	0.302 (0.274,0.331)	0.297 (0.266,0.329)
SC	0.152 (0.128,0.177)	0.189 (0.162,0.216)	0.173 (0.147,0.200)	0.109 (0.076,0.142)	0.197 (0.174,0.219)	0.205 (0.179,0.231)	0.206 (0.177,0.235)	0.210 (0.185,0.236)	0.150 (0.127,0.172)	0.171 (0.144,0.197)	0.170 (0.141,0.199)
UA	0.159 (0.135,0.184)	0.200 (0.173,0.227)	0.185 (0.159,0.211)	0.156 (0.125,0.188)	0.119 (0.096,0.142)	0.125 (0.098,0.151)	0.154 (0.125,0.183)	0.173 (0.147,0.198)	0.145 (0.123,0.167)	0.131 (0.104,0.157)	0.113 (0.083,0.143)
PD	0.242 (0.216,0.267)	0.148 (0.120,0.176)	0.186 (0.158,0.214)	0.237 (0.204,0.271)	0.234 (0.211,0.257)	0.194 (0.167,0.220)	0.171 (0.142,0.201)	0.225 (0.199,0.250)	0.287 (0.263,0.310)	0.214 (0.186,0.241)	0.236 (0.206,0.265)
AD	0.190 (0.165,0.215)	0.146 (0.117,0.174)	0.198 (0.171,0.224)	0.150 (0.117,0.183)	0.201 (0.179,0.224)	0.187 (0.160,0.214)	0.218 (0.188,0.247)	0.142 (0.116,0.168)	0.167 (0.145,0.189)	0.183 (0.156,0.210)	0.184 (0.154,0.214)
L2	0.200 (0.171,0.228)	0.228 (0.194,0.262)	0.239 (0.207,0.270)	0.187 (0.154,0.219)	0.220 (0.194,0.247)	0.194 (0.166,0.222)	0.175 (0.148,0.202)	0.322 (0.287,0.357)	0.215 (0.190,0.240)	0.221 (0.188,0.253)	0.156 (0.128,0.184)
L3	0.353 (0.324,0.381)	0.367 (0.334,0.400)	0.351 (0.319,0.383)	0.271 (0.237,0.305)	0.298 (0.271,0.325)	0.286 (0.258,0.315)	0.211 (0.184,0.239)	0.345 (0.309,0.382)	0.306 (0.280,0.332)	0.344 (0.313,0.376)	0.285 (0.256,0.314)
L4	0.720 (0.690,0.751)	0.699 (0.663,0.734)	0.670 (0.636,0.705)	0.600 (0.563,0.636)	0.726 (0.698,0.755)	0.722 (0.691,0.753)	0.645 (0.616,0.673)	0.837 (0.797,0.878)	0.638 (0.612,0.665)	0.778 (0.743,0.813)	0.703 (0.672,0.733)

Some individual characteristics can be spotted from Fig. 2. South Korea showed the largest difference between the dimensions of mobility and self-care. Japan had similar weights for dimensions other than mobility. Hong Kong, Malaysia and Taiwan showed similar rank order, i.e. Mobility > Pain/discomfort > Anxiety/depression > Self-care > Usual activities. China differed with these three studies by placing usual activities more important than self-care. Indonesia showed a different pattern by weighing more on usual activities and self-care over pain/discomfort and anxiety/depression. Both Vietnam and Singapore had similar weights three dimensions. Thailand and Vietnam were unique in the sense that Thailand valued anxiety/depression as the second most important dimension and Vietnam valued pain/discomfort as the most important dimension.

**Fig. 3:**
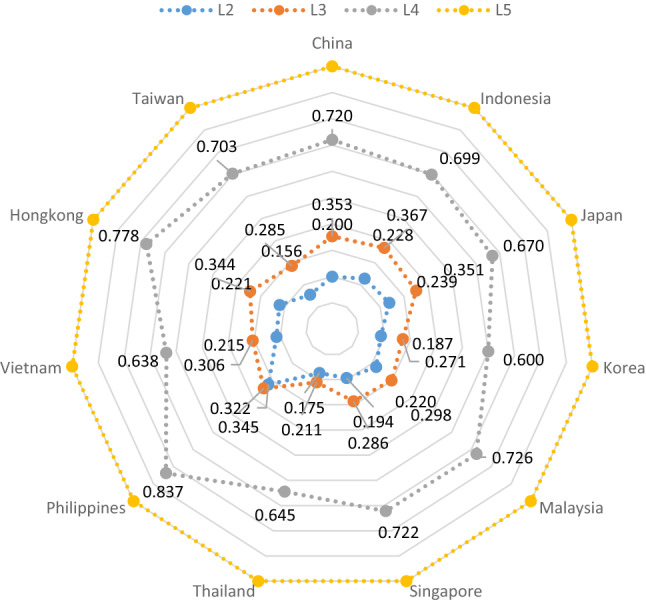
Relative importance of the severity levels

Compared with the large variations among the relative importance of health dimensions, the relative importance of levels were more comparable across studies (Fig. 3). The weights of mild (L2) and moderate problems (L3) were more similar across regions as compared to the weights of severe (L4). The L2 ranges from 0.156 for Taiwan to 0.322 for the Philippines; the L3 ranges from 0.211 for Thailand to 0.367 for Indonesia; the L4 ranges from 0.600 for South Korea to 0.837 for the Philippines. In the Philippines and Thailand, the difference between level 2 and level 3 were minimal. On average, level 2 accounted for 20% of the weight of level 5, level 3 accounted for approximately 30% of the weight of level 5 and level 4 accounted for 70% of the weight of the level 5. The smallest relative importance was 0.156 of L2 from Taiwan, which represents having a mild problem accounted for about 15.6% weight of having an extreme problem. The smallest L3 was from Thailand (0.211), and this value was smaller than L2 from some studies.

## Discussion

The present study compared the DCE based modeling results and relative importance of EQ-5D-5L dimensions and levels of 11 Asian valuation studies. The strength of this study is all 11 studies followed the standardized EQ-VT protocol, which minimized possible noises in identifying the true differences. Based on our results, it is fair to declare that there does not exist a single preference pattern for Asian populations. This is in line with a previous study comparing TTO preference data [10]. A clear distinction between our DCE results and the TTO results is the relative weights for level 3 and level 4 are larger in the TTO study.

Our study first tested the differences of modeling coefficients and then compared the relative importance attached to the dimensions and levels of EQ-5D-5L. Both analyses suggest large health preference heterogeneities among Asians. First, the number of differed coefficients ranged between 2 (Malaysia vs. Taiwan) and 16 (Singapore vs. Malaysia) and the average number is 9.3, suggesting about half of the coefficients differed when pooled two studies’ data for a joint model. Second, both the relative importance of dimensions and levels differed among studies. Only Hong Kong, Taiwan and Malaysia showed the same order of five dimensions. Here we concluded some common patterns that, however, always come with exceptions. First, among the five dimensions, mobility is the most important dimension for every population except for Vietnam. This is similar to the results from a comparison of TTO-only preference data from 7 Asian regions [10]. However, western countries do not value mobility as highly; the Dutch, German, and US populations view mobility as third, fourth, and second most important dimension, respectively [34–36]. Purba et al. argued that in the western developed countries, problems with mobility had less influence due to better infrastructure provision and less emphasis on manual labor [20]. However, in high income and developed regions such as Singapore and Japan, mobility is still the most valued dimension. Second, the sum of three function dimensions (mobility, self-care and usual activities) were higher than the sum of two symptom dimensions (pain/discomfort and anxiety/depression). Also, either usual activities or self-care is the least important dimension. Indonesia and Philippines are the exceptions. This result agrees with the previous study of comparing TTO data among 7 Asian populations. In that study, Indonesia was the only one who valued pain/discomfort and anxiety/depression the lowest. Third, pain/discomfort was valued more important than anxiety/depression and is the second most important dimensions for 6 studies. These characteristics mark some notable difference between preference pattern from most European, American, and African populations [5, 37–39].

Despite these similarities, it is clear that a singular preference pattern does not exist for all Asian populations. For example, there is no agreement on the least important dimension in our comparison: 3 studies valued self-care, 2 studies valued anxiety/depression, and 6 studies valued usual activities as the least important. This contrasts to a previous study of comparing health preference pattern for Canada, England, the Netherlands, and Spain. In that study, Olsen et al. found a clear pattern existed for these four western countries and named it western preference pattern (WePP) [5]. In the WePP, four general characteristics were noticed in terms of the relative importance: 1) (PD + AD) ≈ (MO + SC + UA); 2) PD ≈ AD; 3) MO ≈ SC; 4) UA < SC. However, no Asian preferences fit well with these four characteristics. In fact, the sum of pain/discomfort and anxiety/depression was less than the weight of the other three dimensions in all Asian studies: (PD + AD) < (MO + SC + UA), suggesting that compared with the western countries, the Asian placed more weights on the functional dimensions. The second characteristic of ‘PD ≈ AD’ was only observed in the results from Indonesia, Singapore, and Malaysia. The third characteristic was clearly invalid in Asia as mobility was valued as the most important dimension while self-care had less relative importance in 4 studies. For the last characteristic, four Asian populations put similar or higher values for usual activities.

The differences of health preferences can be attributed to several reasons. First, in our sample, 11 populations come from diverse cultural, economic, political and social environments. Although no study has examined how these factors related to health preferences, country specific value set has been established on the notion that these factors shape people’s preference. Second, even though each study followed the same study protocol, their sampling method differed. Quota sampling method was the most used sampling strategy, but the quota varied across study. For example, ethnicity was used in some studies like Malaysia and Singapore, but not in China and South Korea. Similarly, some studies only recruited participants from urban areas, which may not be representative for the whole target population. Studies have shown that the demographic of respondents could influence the health preferences [40, 41]. Hence, different respondents recruited for each study may contribute to the observed differences. Last but not least, the EQ-5D-5L descriptive system was translated into different official languages from English. Though a standardized translation process was conducted to maintain equivalence between the translated questionnaire and its source version, different languages have different ways of expression which maybe inadequately captured [42].

This study has some limitations. First, the point estimates of the relative weights were used to identify the preference pattern. Considering the 95% confidence intervals were overlapped for some dimensions, the relative weight difference between dimensions may not be statistically significant. Assuming a scale length of 1.5 (i.e. 55555 has a value of -0.5, 11111 has a value of 1) and using a MID of 0.05, any relative importance difference over 0.03 should be meaningful. Nevertheless, since we do not know the actual scale length of each study, we did not use this criterion. Second, even though a standardized protocol was used, the demographic questions used for each study was customized by each local study team. Due to these sampling variations, we did not further test how these variations affect preferences. Only the heteroscedastic model shown in Online Appendix 4 demonstrates that the variances was constant for respondents with different ages and gender.

Norman et al. pointed out that differences in methods obscured the true differences in health preferences across countries after comparing published EQ-5D-3L value sets [6]. Our study has shown that using a standardized data collection protocol, study design and modeling choice, there still remained differences in EQ-5D-5L modeling results and the relative importance of dimensions and levels among Asian populations. Therefore, the effort of estimating a combined continental value set that was carried out for European and Western countries [5, 43] should be discouraged for Asia.

## Conclusion

By comparing the DCE data modeling results, we found that the rank order of EQ-5D-5L dimensions and the relative weight of levels differed among Asian populations. These findings confirmed the health preference heterogeneity among Asian populations that was observed in previous studies using TTO data. All the evidence suggested the necessity of using local value set for estimating health utility.

## Supplementary Information

Below is the link to the electronic supplementary material.

Supplementary file1 (xlsx 26 KB)

Supplementary file2 (docx 19 KB)

Supplementary file3 (docx 23 KB)

Supplementary file4 (docx 26 KB)
