# Supplementary material for: Do health preferences differ among Asian populations? A comparison of EQ-5D-5L discrete choice experiments data from 11 Asian studies
Source: Qual Life Res. 2022 Feb 18;31(7):2175–87. doi: 10.1007/s11136-021-03075-x (PMC9188617; doi:10.1007/s11136-021-03075-x)
Supplement: Supplementary file 2 — Supplementary file2 (docx 19 KB) [file 11136_2021_3075_MOESM2_ESM.docx]

Appendix 2. Example STATA code for calculating the standard errors of the relative importance using the Delta method

Article name: Do health preferences differ among Asian populations? A comparison of EQ-5D-5L Discrete Choice Experiments data from 11 Asian studies

Journal name: Quality of life research

Author names: Zhihao Yang, Fredrick Dermawan Purba, Asrul Akmal Shafie, Ataru Igarashi, Eliza Wong, Hilton Lam, Hoang Van Minh, Hsiang-Wen Lin, Jeonghoon Ahn, Juntana Pattanaphesaj, Min-Woo Jo, Vu Quynh Mai, Jan Busschbach, Nan Luo, Jie Jiang

Affiliation and e-mail address of the corresponding author: Jinan University, jiangjie218@126.om

///Example mixlogit modelling

mixlogit value,group(dce_group_identifier) rand(mo2-ad5) id(Participant_id)

///Using the mixedlogit results, estimate the standard errors for the relative importance of three levels

///For level2

nlcom ((_b[mo2] + _b[sc2] + _b[ua2] + _b[pd2] + _b[ad2])/*

*/ /(_b[mo5] + _b[sc5] + _b[ua5] + _b[pd5] + _b[ad5]))

///For level 3

nlcom ((_b[mo3] + _b[sc3] + _b[ua3] + _b[pd3] + _b[ad3])/*

*/ /(_b[mo5] + _b[sc5] + _b[ua5] + _b[pd5] + _b[ad5]))

///For level 4

nlcom ((_b[mo4] + _b[sc4] + _b[ua4] + _b[pd4] + _b[ad4])/*

*/ /(_b[mo5] + _b[sc5] + _b[ua5] + _b[pd5] + _b[ad5]))

///Using the mixedlogit results, estimate the standard errors for the relative importance of five dimensions

///For MO

nlcom ((_b[mo2])/(_b[mo2] + _b[sc2] + _b[ua2] + _b[pd2] + _b[ad2])/*

*/ +(_b[mo3])/(_b[mo3] + _b[sc3] + _b[ua3] + _b[pd3] + _b[ad3])/*

*/+(_b[mo4])/(_b[mo4] + _b[sc4] + _b[ua4] + _b[pd4] + _b[ad4])/*

*/+(_b[mo5])/(_b[mo5] + _b[sc5] + _b[ua5] + _b[pd5] + _b[ad5]))/4

///For SC

nlcom ((_b[sc2])/(_b[mo2] + _b[sc2] + _b[ua2] + _b[pd2] + _b[ad2])/*

*/ +(_b[sc3])/(_b[mo3] + _b[sc3] + _b[ua3] + _b[pd3] + _b[ad3])/*

*/+(_b[sc4])/(_b[mo4] + _b[sc4] + _b[ua4] + _b[pd4] + _b[ad4])/*

*/+(_b[sc5])/(_b[mo5] + _b[sc5] + _b[ua5] + _b[pd5] + _b[ad5]))/4

///For UA

nlcom ((_b[ua2])/(_b[mo2] + _b[sc2] + _b[ua2] + _b[pd2] + _b[ad2])/*

*/ +(_b[ua3])/(_b[mo3] + _b[sc3] + _b[ua3] + _b[pd3] + _b[ad3])/*

*/+(_b[ua4])/(_b[mo4] + _b[sc4] + _b[ua4] + _b[pd4] + _b[ad4])/*

*/+(_b[ua5])/(_b[mo5] + _b[sc5] + _b[ua5] + _b[pd5] + _b[ad5]))/4

///For PD

nlcom ((_b[pd2])/(_b[mo2] + _b[sc2] + _b[ua2] + _b[pd2] + _b[ad2])/*

*/ +(_b[pd3])/(_b[mo3] + _b[sc3] + _b[ua3] + _b[pd3] + _b[ad3])/*

*/+(_b[pd4])/(_b[mo4] + _b[sc4] + _b[ua4] + _b[pd4] + _b[ad4])/*

*/+(_b[pd5])/(_b[mo5] + _b[sc5] + _b[ua5] + _b[pd5] + _b[ad5]))/4

///For AD

nlcom ((_b[ad2])/(_b[mo2] + _b[sc2] + _b[ua2] + _b[pd2] + _b[ad2])/*

*/ +(_b[ad3])/(_b[mo3] + _b[sc3] + _b[ua3] + _b[pd3] + _b[ad3])/*

*/+(_b[ad4])/(_b[mo4] + _b[sc4] + _b[ua4] + _b[pd4] + _b[ad4])/*

*/+(_b[ad5])/(_b[mo5] + _b[sc5] + _b[ua5] + _b[pd5] + _b[ad5]))/4
