# Supplementary material for: Do health preferences differ among Asian populations? A comparison of EQ-5D-5L discrete choice experiments data from 11 Asian studies
Source: Qual Life Res. 2022 Feb 18;31(7):2175–87. doi: 10.1007/s11136-021-03075-x (PMC9188617; doi:10.1007/s11136-021-03075-x)
Supplement: Supplementary file 3 — Supplementary file3 (docx 23 KB) [file 11136_2021_3075_MOESM3_ESM.docx]

Appendix 3. An example of testing coefficients difference using mixed logit model: China vs Indonesia

Article name: Do health preferences differ among Asian populations? A comparison of EQ-5D-5L Discrete Choice Experiments data from 11 Asian studies

Journal name: Quality of life research

Author names: Zhihao Yang, Fredrick Dermawan Purba, Asrul Akmal Shafie, Ataru Igarashi, Eliza Wong, Hilton Lam, Hoang Van Minh, Hsiang-Wen Lin, Jeonghoon Ahn, Juntana Pattanaphesaj, Min-Woo Jo, Vu Quynh Mai, Jan Busschbach, Nan Luo, Jie Jiang

Affiliation and e-mail address of the corresponding author: Jinan University, jiangjie218@126.om

| Main effects | Coefficients, SE | SD coefficients, SE | Main effects*study dummy | Coefficients, SE |  |
| --- | --- | --- | --- | --- | --- |
| mo2 | -0.519,0.058 | **0.060**,0.168 | mo2*Indonesia | **-0.093**,0.086 |  |
| mo3 | -0.913,0.074 | 0.353,0.132 | mo3*Indonesia | **-0.072**,0.105 |  |
| mo4 | -1.552,0.078 | 0.403,0.138 | mo4*Indonesia | -0.339,0.108 |  |
| mo5 | -2.356,0.100 | 1.118,0.097 | mo5*Indonesia | -0.634,0.132 |  |
| sc2 | -0.219,0.064 | **0.023**,0.161 | sc2*Indonesia | -0.242,0.095 |  |
| sc3 | -0.491,0.072 | 0.317,0.142 | sc3*Indonesia | **-0.172**,0.106 |  |
| sc4 | -1.089,0.076 | -0.358,0.179 | sc4*Indonesia | **0.061**,0.108 |  |
| sc5 | -1.479,0.078 | 0.550,0.103 | sc5*Indonesia | **-0.021**,0.107 |  |
| ua2 | -0.350,0.062 | **0.153**,0.171 | ua2*Indonesia | **-0.044**,0.092 |  |
| ua3 | -0.450,0.070 | **0.155**,0.137 | ua3*Indonesia | **-0.205**,0.105 |  |
| ua4 | -0.965,0.074 | 0.418,0.127 | ua4*Indonesia | -0.374,0.106 |  |
| ua5 | -1.618,0.081 | 0.712,0.090 | ua5*Indonesia | **-0.134**,0.111 |  |
| pd2 | -0.518,0.065 | **-0.101**,0.147 | pd2*Indonesia | **0.165**,0.095 |  |
| pd3 | -0.742,0.070 | **0.000**,0.132 | pd3*Indonesia | 0.325,0.101 |  |
| pd4 | -1.675,0.080 | 0.459,0.119 | pd4*Indonesia | 0.864,0.108 |  |
| pd5 | -2.059,0.088 | -0.632,0.120 | pd5*Indonesia | 0.974,0.112 |  |
| ad2 | -0.230,0.069 | 0.439,0.105 | ad2*Indonesia | **0.011**,0.101 |  |
| ad3 | -0.691,0.069 | **0.157**,0.110 | ad3*Indonesia | **0.185**,0.101 |  |
| ad4 | -1.453,0.080 | **-0.296**,0.161 | ad4*Indonesia | 0.424,0.108 |  |
| ad5 | -1.837,0.087 | -0.659,0.106 | ad5*Indonesia | 0.421,0.116 |  |
| Number of observations 32,956  Log-likelihood -8587.43 | | | | |  |

*Bold font: not significant at 0.05 level

Appendix 3 shows the pooled modeling of China and Indonesia. The fourth column shows the additional 20 parameters representing the differences between China’s and Indonesia’s modelling results. For example, for mobility, the 4^th^ and 5^th^ levels are significant at 0.05 level and suggests the Indonesian population attached larger disutility to the 4^th^ (0.339) and 5^th^ (0.634) level problems compared with the Chinese population.
