# Supplementary material for: Do health preferences differ among Asian populations? A comparison of EQ-5D-5L discrete choice experiments data from 11 Asian studies
Source: Qual Life Res. 2022 Feb 18;31(7):2175–87. doi: 10.1007/s11136-021-03075-x (PMC9188617; doi:10.1007/s11136-021-03075-x)
Supplement: Supplementary file 4 — Supplementary file4 (docx 26 KB) [file 11136_2021_3075_MOESM4_ESM.docx]

Appendix 4. The results of the heteroscedastic model.

Article name: Do health preferences differ among Asian populations? A comparison of EQ-5D-5L Discrete Choice Experiments data from 11 Asian studies

Journal name: Quality of life research

Author names: Zhihao Yang, Fredrick Dermawan Purba, Asrul Akmal Shafie, Ataru Igarashi, Eliza Wong, Hilton Lam, Hoang Van Minh, Hsiang-Wen Lin, Jeonghoon Ahn, Juntana Pattanaphesaj, Min-Woo Jo, Vu Quynh Mai, Jan Busschbach, Nan Luo, Jie Jiang

Affiliation and e-mail address of the corresponding author: Jinan University, jiangjie218@126.om

| Appendix 4. Modelling results of the heteroscedastic conditional logit model (coefficient, standard error) | | | | | | | | | | | |
| --- | --- | --- | --- | --- | --- | --- | --- | --- | --- | --- | --- |
|  | China | Indonesia | Japan | South Korea | Malaysia | Singapore | Thailand | Philippine | Vietnam | Hong Kong | Taiwan |
| **Explanatory Variables*** |  |  |  |  |  |  |  |  |  |  |  |
| mo2 | -0.477, 0.070 | -0.399, 0.059 | -1.153, 0.152 | -0.553, 0.083 | -0.520, 0.073 | -0.430, 0.054 | -0.464, 0.074 | -0.676, 0.100 | -0.575, 0.074 | -0.582, 0.087 | -0.711, 0.104 |
| mo3 | -0.796, 0.097 | -0.624, 0.082 | -1.430, 0.185 | -0.669, 0.100 | -0.782, 0.096 | -0.568, 0.066 | -0.479, 0.084 | **-0.586**, 0.100 | -0.603, 0.083 | -0.887, 0.116 | -1.076, 0.145 |
| mo4 | -1.367, 0.141 | -1.185, 0.132 | -2.447, 0.263 | -1.144, 0.148 | -1.590, 0.151 | -1.193, 0.105 | -1.289, 0.146 | -1.476, 0.180 | -1.378, 0.141 | -1.662, 0.187 | -1.971, 0.222 |
| mo5 | -2.021, 0.196 | -1.814, 0.195 | -3.532, 0.357 | -2.034, 0.249 | -2.295, 0.205 | -1.711, 0.144 | -2.536, 0.264 | -2.021, 0.237 | -2.382, 0.232 | -2.338, 0.250 | -2.803, 0.309 |
| sc2 | -0.173, 0.063 | -0.287, 0.055 | -0.583, 0.125 | **-*0.098***, 0.058 | -0.399, 0.071 | -0.225, 0.049 | -0.370, 0.072 | -0.503, 0.087 | -0.311, 0.065 | -0.262, 0.068 | -0.293, 0.088 |
| sc3 | -0.420, 0.075 | -0.394, 0.066 | -0.763, 0.142 | -0.138, 0.063 | -0.495, 0.080 | -0.389, 0.059 | **-0.355**, 0.077 | -0.541, 0.095 | **-0.288**, 0.070 | -0.374, 0.077 | -0.509, 0.104 |
| sc4 | -0.955, 0.110 | -0.647, 0.081 | -1.508, 0.184 | -0.387, 0.074 | -1.376, 0.134 | -0.908, 0.087 | -1.108, 0.129 | -1.331, 0.167 | -0.898, 0.105 | -1.116, 0.133 | -1.363, 0.168 |
| sc5 | -1.306, 0.139 | -0.916, 0.106 | -2.167, 0.232 | -0.706, 0.100 | -1.624, 0.150 | -1.153, 0.104 | -1.501, 0.161 | -1.494, 0.178 | -1.381, 0.144 | -1.375, 0.155 | -1.767, 0.207 |
| ua2 | -0.349, 0.068 | -0.269, 0.052 | -0.744, 0.127 | -0.220, 0.059 | -0.285, 0.064 | -0.211, 0.047 | -0.292, 0.066 | -0.447, 0.083 | -0.263, 0.061 | -0.256, 0.066 | -0.171, 0.080 |
| ua3 | -0.413, 0.075 | -0.412, 0.065 | -0.898, 0.143 | -0.243, 0.065 | -0.329, 0.072 | -0.250, 0.053 | -0.336, 0.074 | -**0.439**, 0.088 | -0.318, 0.068 | -0.361, 0.076 | -0.312, 0.094 |
| ua4 | -0.877, 0.105 | -0.858, 0.099 | -1.844, 0.206 | -0.633, 0.094 | -0.955, 0.104 | -0.609, 0.069 | -0.907, 0.111 | -1.072, 0.143 | -1.050, 0.116 | -0.912, 0.114 | -1.271, 0.162 |
| ua5 | -1.448, 0.151 | -1.121, 0.123 | -2.603, 0.267 | -1.122, 0.141 | -1.220, 0.120 | -0.778, 0.080 | -1.566, 0.168 | -1.304, 0.161 | -1.773, 0.177 | -1.156, 0.136 | -1.810, 0.213 |
| pd2 | -0.468, 0.075 | -0.226, 0.053 | -0.633, 0.130 | -0.280, 0.066 | -0.530, 0.077 | -0.241, 0.049 | -0.261, 0.068 | -0.564, 0.094 | -0.637, 0.084 | -0.386, 0.075 | -0.497, 0.097 |
| pd3 | -0.682, 0.092 | -0.257, 0.056 | -0.937, 0.151 | -0.422, 0.078 | -0.595, 0.087 | -0.254, 0.053 | -0.307, 0.074 | -0.594, 0.098 | -0.983, 0.111 | -0.600, 0.092 | -0.673, 0.113 |
| pd4 | -1.501, 0.158 | -0.505, 0.073 | -1.922, 0.225 | -1.063, 0.138 | -1.532, 0.147 | -0.932, 0.089 | -1.186, 0.135 | -1.450, 0.175 | -1.588, 0.162 | -1.304, 0.149 | -1.746, 0.208 |
| pd5 | -1.797, 0.184 | -0.652, 0.084 | -3.014, 0.309 | -1.483, 0.185 | -2.162, 0.198 | -1.388, 0.122 | -1.505, 0.161 | -1.609, 0.191 | -2.138, 0.211 | -1.476, 0.164 | -2.683, 0.306 |
| ad2 | -0.225, 0.068 | -0.139, 0.050 | -0.560, 0.127 | -0.121, 0.059 | -0.415, 0.075 | -0.215, 0.051 | -0.302, 0.072 | -0.270, 0.075 | -0.302, 0.067 | -0.318, 0.074 | -0.189, 0.088 |
| ad3 | -0.623, 0.089 | -0.319, 0.058 | -1.164, 0.162 | -0.314, 0.069 | -0.609, 0.084 | -0.362, 0.057 | -0.520, 0.084 | -0.436, 0.086 | -0.627, 0.086 | -0.531, 0.089 | -0.780, 0.125 |
| ad4 | -1.283, 0.144 | -0.641, 0.083 | -2.052, 0.231 | -0.680, 0.101 | -1.401, 0.138 | -0.952, 0.093 | -1.460, 0.159 | -1.001, 0.132 | -1.023, 0.117 | -1.205, 0.148 | -1.656, 0.208 |
| ad5 | -1.619, 0.173 | -0.885, 0.105 | -3.132, 0.323 | -0.873, 0.120 | -1.922, 0.177 | -1.231, 0.112 | -2.055, 0.213 | -1.012, 0.136 | -1.354, 0.142 | -1.393, 0.163 | -2.186, 0.261 |
| **Heteroscedastic Variables#** |  |  |  |  |  |  |  |  |  |  |  |
| Age | 0, 0.001 | 0.001, 0.002 | -0.016, 0.001 | 0.001, 0.002 | 0.002, 0.001 | 0.001, 0.001 | 0.001, 0.002 | 0.001, 0.002 | 0.001, 0.002 | -0.003, 0.001 | -0.002, 0.002 |
| Gender | -0.072, 0.045 | 0.154, 0.052 | 0.037, 0.050 | -0.020, 0.053 | -0.081, 0.046 | -0.046, 0.041 | -0.065, 0.046 | -0.102, 0.053 | -0.005, 0.046 | 0.083, 0.052 | -0.083, 0.049 |
| Number of observations | 18,200 | 14,756 | 16,850 | 15,120 | 16,002 | 25,886 | 16,898 | 14,000 | 16,814 | 14,196 | 14,000 |
| Log-likelihood | -4721.89 | -3954.10 | -4529.14 | -4148.22 | -3784.79 | -7194.25 | -4112.91 | -3794.87 | -4017.65 | -3647.90 | -3283.18 |
| AIC | 9487.78 | 7952.19 | 9102.28 | 8340.45 | 7613.59 | 14432.51 | 8269.81 | 7633.74 | 8079.30 | 7339.80 | 6610.36 |
| BIC | 9659.58 | 8119.38 | 9272.39 | 8508.17 | 7782.56 | 14612.06 | 8439.98 | 7799.77 | 8249.36 | 7506.13 | 6776.39 |

* For the explanatory variables: italic and bold font suggests the coefficient is not significant at 0.05 level; bold font suggests the coefficient is inconsistent.

#For the Heteroscedastic Variables: all coefficients were not significant except the gender variable for Japan.

Appendix 4 shows the modelling results of the Heteroscedastic model (‘clogithet’ in STATA). The two heteroscedastic variables show whether the variances vary between these two variables, namely age and gender. Only the gender variable in Japan was significant at 0.05 level suggesting the variances is not constant between gender subgroups in Japan.
